# Case Report: Acupuncture for acute colonic pseudo- obstruction in a 90-year-old patient with COVID-19 infection

**DOI:** 10.3389/fmed.2025.1676907

**Published:** 2025-12-30

**Authors:** Binjun Liao, Meng Ren, Yang Zhao, Jun Rong

**Affiliations:** 1 Department of Rehabilitation, Hangzhou Red Cross Hospital, Hangzhou, China; 2 Rainbowfish Rehabilitation and Nursing School, Hangzhou Polytechnic University, Hangzhou, China

**Keywords:** acupuncture, acute colonic pseudo-obstruction, COVID-19, elderly patient, case report

## Abstract

**Introduction:**

Acute colonic pseudo-obstruction (ACPO) is a severe gastrointestinal motility disorder defined by significant dilation of the colon without any mechanical blockage. In recent years, studies have shown that ACPO, as a rare complication of severe COVID-19, requires prolonged ICU treatment and still carries an extremely high risk. Recent reports indicate that patients with severe COVID-19 are at an increased risk of developing complications, including ACPO. These patients often require extended treatment in the intensive care unit and may undergo multiple drug therapies. Despite aggressive treatment efforts, their mortality risk remains elevated.

**Methods:**

We use the acupuncture points selected under the guidance of traditional Chinese medicine theory, combined with the application of 5 Hz electroacupuncture.

**Results:**

In a 90-year-old male, severe COVID-19 developed acute colonic pseudo-obstruction (ACPO). Despite fasting, gastrointestinal decompression, enema, and other life support measures, ACPO further deteriorated. Six days after admission, the patient’s abdominal girth has increased to 93 cm. At last, we decided to use acupuncture. One hour after acupuncture, the patient began to defecate. From then on, acupuncture treatment was carried out daily, and the patient defecated regularly. On the third day after the acupuncture treatment, the patient began to receive a small amount of enteral nutrition. Nine days after starting acupuncture, a CT scan confirmed that there was no intestinal obstruction.

**Conclusion:**

Early acupuncture intervention appears safe and effective for ACPO, particularly in elderly patients with surgical contraindications. This case suggests that pattern differentiation-guided point selection and frequency-specific electroacupuncture are critical determinants of therapeutic efficacy.

## 1 Introduction

**FIGURE 1:**
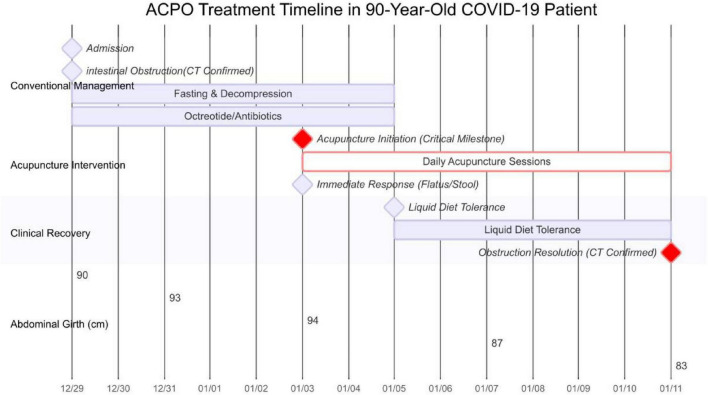
Overview of key events and clinical changes in the treatment of this case.

Acute colonic pseudo-obstruction (ACPO), also known as Ogilvie’s syndrome, includes imaging evidence of colonic dilation ≥ 9 cm without a physical obstruction, occurring primarily in patients with severe comorbidities (1, 2). People over the age of 60 are at a high risk. Infections, trauma, or surgical procedures are typical triggers (3). It is reported that the novel coronavirus may cause severe complications, including ACPO (4). The mechanism might involve ACE2 receptors on the surface of small intestinal cells, which mediate the virus’ invasion, amplification, and activation of gastrointestinal inflammation (5, 6). ACPO is a serious condition often requiring prompt medical intervention. Initial conservative management includes fasting, correction of electrolyte abnormalities, fluid resuscitation, treatment of infections, gastrointestinal decompression, enemas, and bed rest exercise to promote GI activity. Neostigmine enhances intestinal motility but may cause bradycardia and hypotension. Invasive procedures, used when conservative measures fail, carry risks of infection and perforation. This case report discusses a nonagenarian patient who developed ACPO following a COVID-19 infection. On Day 1, the patient was admitted with intestinal obstruction by CT, due to the patient’s advanced age and preference for conservative treatment. Day 6, the ACPO symptoms showed no significant improvement. Acupuncture was then introduced. After 1 h of acupuncture, the patient began to defecate. On Day 8 (On the third day of the acupuncture treatment), the patient began receiving a small amount of enteral nutrition. On Day 14 (9 days after the start of acupuncture treatment), a CT scan confirmed the absence of intestinal obstruction (Figure 1).

This case represents the first documented resolution of COVID-19-associated ACPO using acupuncture in a 90-year-old who cannot undergo surgery. Demonstrates unprecedented therapeutic velocity: defecation within 1 h and complete radiological resolution at day 8 [versus 24.6-days average recovery (4)]. Establishes a pattern differentiation-guided protocol integrating Qi-tonifying acupoints with 5 Hz electroacupuncture as a replicable model for geriatric gastrointestinal emergencies. This case complied with the 2013 CARE checklist (see Supplementary material).

## 2 Patient information and conventionl management

**FIGURE 2:**
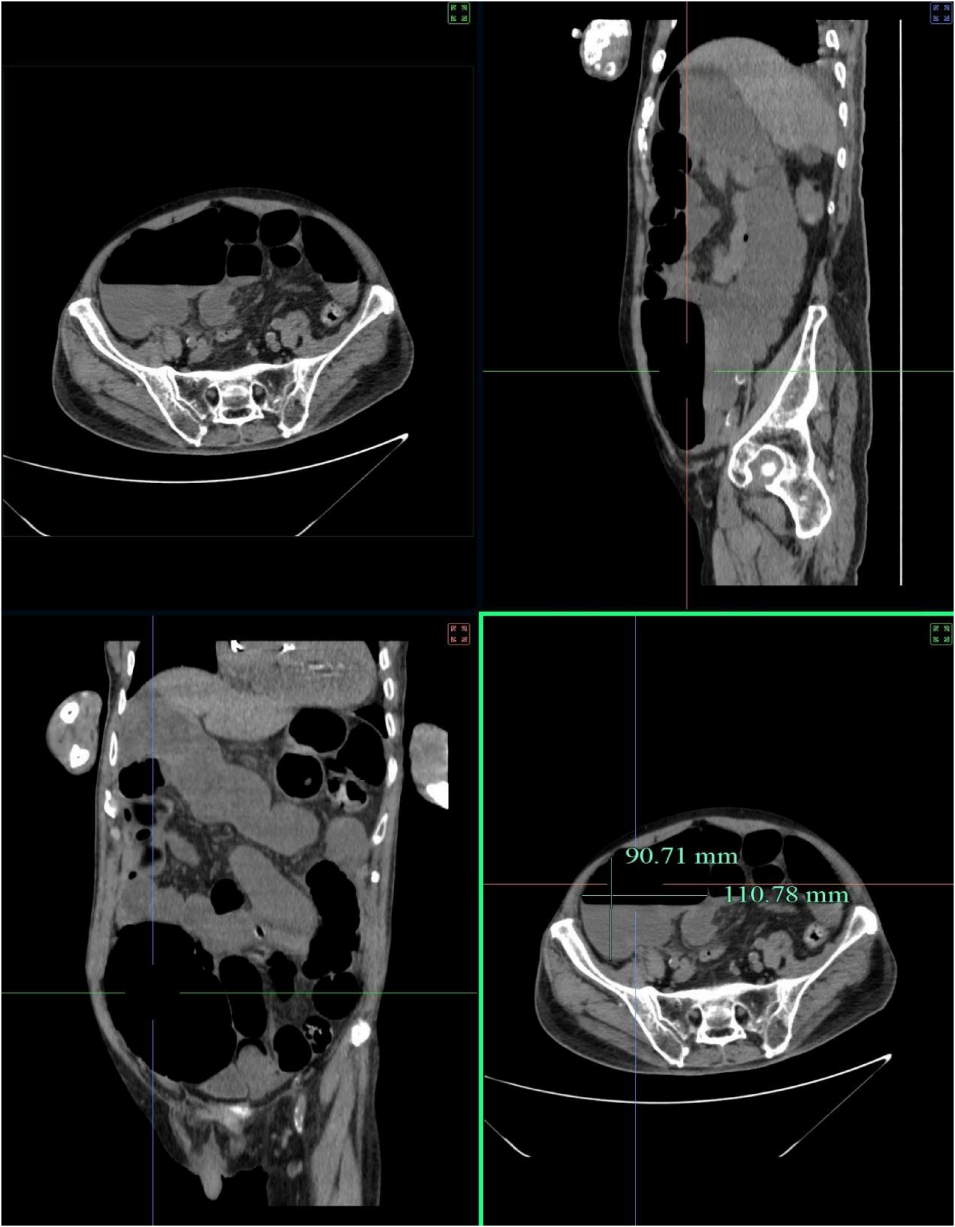
Admission sagittal CT of the abdomen demonstrating intestinal obstruction, characterized by multiple air-fluid levels within dilated bowel loops and marked abdominal distension. The widest part of the horizontal plane was 90.71 mm × 110.78 mm.

A 90-years-old male COVID-19 patient was admitted to the hospital on December 29, 2022. In addition to fever and respiratory symptoms, he presented with abdominal distension, failure to pass flatus or stool for 48 h, and reduced food intake, accompanied by a desire to vomit. The circumference of his abdomen was measured at 90 cm. An initial abdominal scan revealed evidence of low intestinal obstruction that colon and small intestine show marked dilation with fluid accumulation; no definite mass lesion is identified. The widest portion of the colon at the horizontal level measures 90.71 mm × 110.78 mm (Figure 2). Blood pressure upon admission was 90/58 mmHg, Hemoglobin: 87 g/L, Platelets: 92/L, Sodium: 135 mmol/L, C-reactive protein (hs-CRP): 47.32 mg/L, Serum creatinine was 190 umol/l, and the glomerular filtration rate was 27.69 mL/min calculated according to the MDRD formula. Quantitative determination of B-type natriuretic peptide (BNP): 505 pg/ml. His medical history included 20 years of type 2 diabetes (managed with insulin), 2 years of chronic heart failure (treated with sacubitril/valsartan, furosemide, spironolactone), 2 years of benign prostatic hyperplasia (treated with finasteride, doxazosin), and gastrointestinal ulcers (treated with rabeprazole). He had a pacemaker implanted 11 years ago and had a documented penicillin allergy. Significantly, the patient had a 5-years history of chronic constipation.

Following multidisciplinary consultation, the initial treatment prioritized management of intestinal obstruction alongside guideline-directed COVID-19 therapy. The conservative regimen included: intravenous nutritional support, nil *per os*, nasogastric decompression, octreotide (0.3 mg subcutaneously every 12 h), antibiotics (2.0 g Cefoperazone and sulbactam sodium every 12 h), proton pump inhibitor therapy, glycerin enemas, and continuous monitoring of vital signs, electrolytes, and arterial blood gases. Use Nematinze Tablets/Ritonavir Tablets to slow down the replication of the novel coronavirus, use low-dose hormones to alleviate lung inflammation, and low-dose anticoagulant drugs to prevent thrombosis formation. Given the patient’s advanced age, hypotension, and history of gastric ulcers, the family refused to use neostigmine and to insert a drainage tube. Despite conservative management, symptoms persisted and did not improve over 72 h, with abdominal girth increasing from 90 to 93 cm by January 3, 2023.

## 3 Acupuncture treatment

Given the patient’s refractory symptoms despite conventional management and advanced age, acupuncture was initiated as adjunctive therapy. Traditional Chinese Medicine (TCM) assessment identified a dual pattern of Qi-Blood deficiency and intestinal Qi stagnation. The treatment strategy focused on triple objectives: tonifying foundational Qi, invigorating Blood circulation, and resolving intestinal stagnation.

Acupoints were identified based on WHO’s Standard Acupuncture Locations, including Zhongwan (CV12), Xiawan (CV10), and Qihai (CV6) on the midline, and Tianshu (ST25), Daheng (SP15), Zusanli (ST36), Shangjuxu (ST37), Sanyinjiao (SP6), and Taichong (LR3) bilaterally. A bilateral 5 Hz electrical current was administered to the ST36 and ST37 acupoints for a duration of 30 min utilizing an electrical stimulator (SDZ-V, Huatuo, Suzhou, China). Electroacupuncture was administered once a day. In this case, the treatment lasted for a total of 9 days.

## 4 Outcomes

**FIGURE 3:**
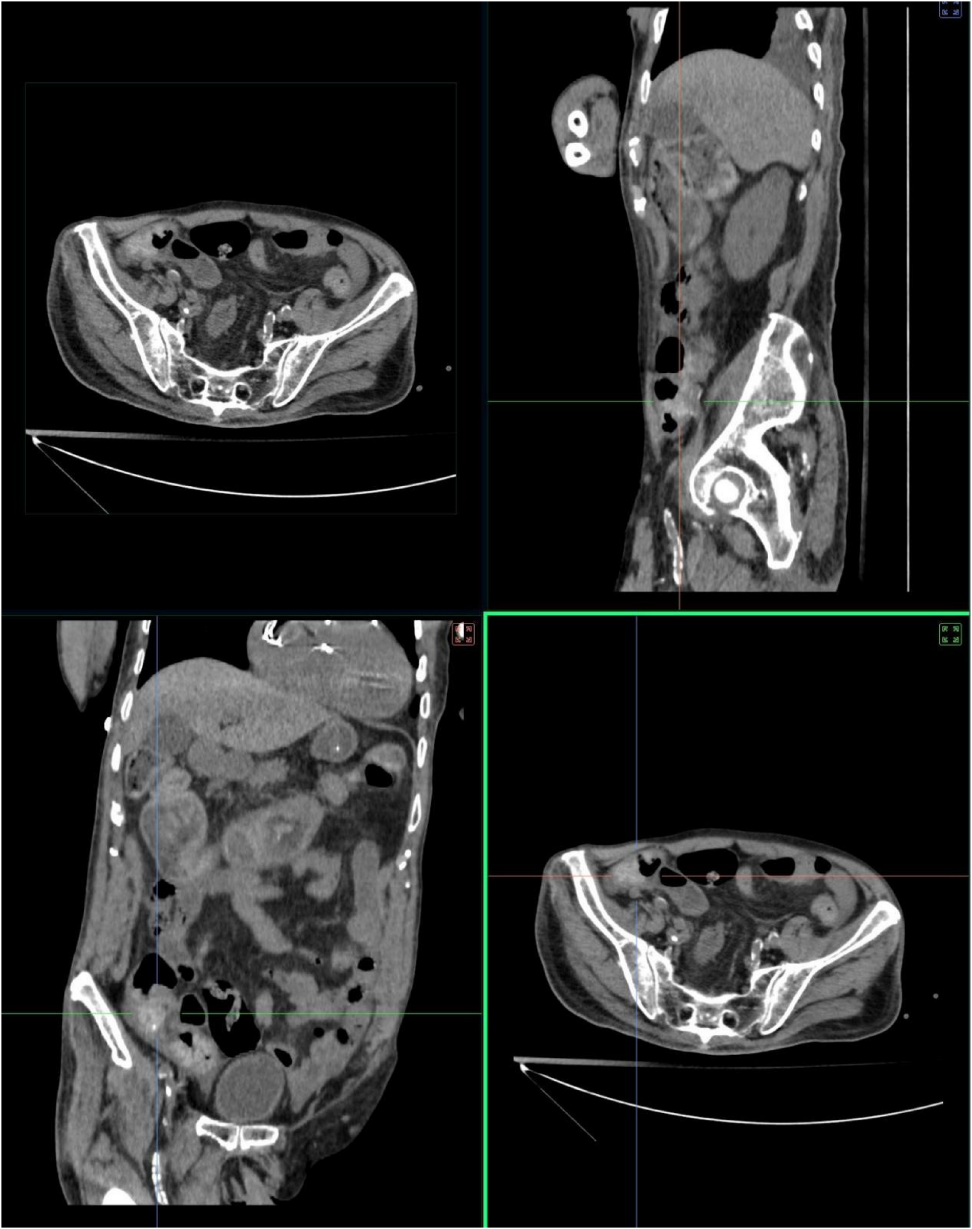
One week after the acupuncture intervention, sagittal CT of the abdomen demonstrating resolution of intestinal obstruction and significant reduction in abdominal distension.

After about 20 min of needle retention during acupuncture, a bowel sound was heard. Within 60 min post-acupuncture, the patient passed flatus followed by a large loose stool (approximately 200 g). Daily acupuncture sessions were subsequently maintained, resulting in the restoration of regular bowel movements and a reduction of abdominal girth to 87 cm. By January 5, 2023, enteral nutrition was cautiously advanced to include small-volume liquid diets. Follow-up abdominal imaging on January 11, 2023, confirmed complete resolution of intestinal obstruction (Figure 3). The improvement appeared to be temporally associated with acupuncture.

## 5 Discussion

Acute colonic pseudo-obstruction carries substantial mortality, particularly among elderly patients with COVID-19 infection (4, 7). Studies report approximately 20% mortality in non-surgically managed ACPO patients aged 51–66 years, with an average pseudo-obstructive symptom duration of 24.6 days (4). To our knowledge, this represents the first documented case of acupuncture resolving COVID-19-associated intestinal obstruction and the oldest reported survivor. Zhao et al. treated an elderly patient with electroacupuncture to improve intestinal obstruction caused by sepsis, who was also an elderly patient (8). Both cases were treated with acupuncture when conventional treatment was ineffective, and both achieved significant improvement in about 1 week. There are some differences in the acupoints, but they are all abdominal and leg acupoints combined. The most significant difference is the depth of acupuncture at abdominal acupoints: in our case, shallow stimulation was used at abdominal acupoints with a depth of about 3 mm, while in Zhao’s case, deep stimulation was used at the abdomen with a depth of 55–60 mm. At the same time, the waveforms we selected for electroacupuncture were all sparse waves, differing only by 5 and 20 Hz. Notably, defecation occurred within 60 min post-acupuncture, with complete radiological resolution confirmed by CT at 14 days (Figure 3) – a markedly accelerated recovery compared to the 24.6 days average. This case highlights acupuncture’s potential as an effective adjunct therapy for ACPO, demonstrating both rapid symptomatic relief and sustained anatomical recovery. In addition to this patient, we have also used this prescription to treat many paralytic intestinal obstructions of the qi deficiency type. In the future, further multicenter clinical studies can be carried out to verify its efficacy.

Although the pathophysiology of COVID-19-induced ACPO remains incompletely elucidated, gastrointestinal autonomic nervous system dysfunction is implicated as a pivotal mechanism (9). Research indicates acupuncture modulates gut-brain neural circuits through somatic acupoint stimulation, rebalancing sympathetic-parasympathetic activity to normalize gastrointestinal motility (10). Electroacupuncture at specific points regulates autonomic balance (vagal-sympathetic activity), effectively reversing drug-induced constipation in preclinical models (11). We therefore hypothesize that the immediate therapeutic effect observed – evidenced by defecation within 1 h – resulted from enhanced gastrointestinal autonomic function. This is mechanistically supported by our selection of 5 Hz continuous wave stimulation, which aligns with evidence that low-frequency electroacupuncture optimally modulates vagal neuro-immunological pathways and enhances colonic motility (12). The rapid clinical response (flatus within 60 min) likely reflects ST36/ST37-mediated neuromodulation of jejuno-ileal peristalsis via cholinergic activation (13). A previous review indicated that acupuncture could influence sympathetic and vagal nerve pathways involved in gastric regulation (14). EA at ST36 prevents intestinal inflammation and dysmotility through a neural circuit that requires vagal innervation (15). Concurrently, acupuncture’s long-term effects may involve restoration of gut microbiota composition and metabolic pathways, attenuating immune-inflammatory responses as demonstrated in IBD research (16, 17).

Within the TCM framework, pattern differentiation identified Qi-Blood deficiency, with Qi-tonifying needling techniques specifically addressing deficiency patterns. This individualized approach, while demonstrating significant efficacy, presents challenges for treatment standardization. Future research could leverage artificial intelligence to analyze objective datasets of TCM patterns, establishing evidence-based correlations with optimal acupoint selection, manipulation techniques, and electroacupuncture parameters.

Early acupuncture intervention appears safe and effective for ACPO, particularly in elderly patients with surgical contraindications. This case suggests that pattern differentiation-guided point selection and frequency-specific electroacupuncture are critical determinants of therapeutic efficacy. Although this and other published cases demonstrate that acupuncture can improve functional bowel obstruction, the limitations of individual cases should be considered. Furthermore, concerning the limitations of this case report, it is currently not possible to rule out the involvement of additional factors. Advancing into the big-data era, further clinical studies are warranted to standardize TCM pattern classification and facilitate protocol optimization.

## Data Availability

The original contributions presented in this study are included in this article/Supplementary material, further inquiries can be directed to the corresponding authors.
